# Accelerating the tuning process for optimizing DNN operators by ROFT model

**DOI:** 10.1038/s41598-025-20139-x

**Published:** 2025-10-17

**Authors:** ZiChuan He, Hui Zhong, XiaoHua Shi, ChangHai Zhao, JiaMin Wen, MinQiang Shang

**Affiliations:** 1 School of Software, Beihang University, Beijing, 100083 China; 2 China National Petroleum Corporation, BGP Inc., Zuozhou, 072751 China

**Keywords:** Computational science, Computer science, Software

## Abstract

Deep neural networks (DNNs) are computationally intensive and optimized in different ways. Some compiler optimizations for DNNs could achieve performance almost the same as, or even better than, manual optimizations. However, the former mechanisms usually require an unbearably long optimization time in the tuning process. In this paper, we propose a new method that accelerates the tuning process significantly without performance penalties. In particular, we use a Roofline-like cost model, namely ROFT (Roofline for Fast AutoTune), to evaluate the performance of schedules. The ROFT model can be easily implemented on different microarchitectures, e.g., NVidia GPUs and Huawei Ascend NPUs. Based on the cost model, we implement a flexible two-stage search algorithm, which significantly improves the time of tuning process. Experiments show that the ROFT method speeds up the tuning process by about 4X and 10X compared with AutoTVM on NVidia GPUs and the AutoTune of Huawei’s Tensor Boost Engine (TBE) on Huawei Ascend310 NPUs for some typical DNNs, respectively. It improves the inference time of some DNNs by up to 7% as well.

## Introduction

In recent years, the depth and scale of DNNs have experienced exponential growth. Therefore, researchers are focusing on how to improve the performance of deep neural networks, primarily approaching it from two points of view. The first strategy involves the manual creation and utilization of operator libraries, exemplified by NVidia CuDNN^1^, which is a dedicated optimization library for convolutional operations, and CuBLAS, which is a finely tuned linear algebra library for general matrix multiplication (GEMM) operations. The second strategy focuses on minimizing runtime overheads through the adoption of DNN compilers such as TVM^2^, Tensor Comprehensions^3^, Glow^4^, nGraph^5^, and XLA, etc. These compilers are adept at producing optimized computation graphs and operators for various microarchitectures, including CPUs, GPUs, and NPUs. Among these, AutoTVM stands out by employing a learn-to-compile approach, demonstrating significant performance improvements for operators with static shapes compared to those in traditional operator libraries. Recent trends indicate a growing inclination among researchers to integrate automatic scheduling with manual optimization techniques^6,7^, with the aim of further accelerating model inference speeds, as evidenced by emerging research in this domain.

Using specified schedule templates, the learning-to-compile methodology is capable of generating a myriad of code versions for tensor programs with different combinations of scheduling parameters (also called knobs) during the automatic tuning process. For certain critical operators, such as convolutions, the potential schedule space for code variations can increase to the magnitude of $10^{9}$. Given this vastness, it is clear that a mere random search algorithm would be inadequate to identify efficient optimizations within a reasonable timeframe. To counteract this challenge, AutoTVM integrates a sophisticated search strategy that combines the predictive power of XGBoost^8^ with the optimization efficiency of a simulated annealing (SA) algorithm, thus enhancing the speed of the tuning procedure. Similarly, the Huawei Tensor Boost Engine (TBE) AutoTune employs a Genetic Algorithm (GA) approach, strategically bypassing exhaustive exploration of the entire optimization landscape. Recent advances in the field have seen Wu et al.^9^ introduce the ytopt auto-tuning framework, which adopts Bayesian optimization for a more effective exploration of the parameter space, with the aim of further refine the auto-tuning process. Currently, Robert et al.^10^ have developed an innovative online autotuner that utilizes black-box optimization techniques to expedite the autotuning procedure, marking a significant step towards optimizing computational efficiency in deep learning endeavors.

However, when generating highly optimized code for DNN operators, the learning-to-compile approach may still suffer from an extremely long optimization time. For example, the optimization time of AutoTVM or AutoTune could be dozens of hours for DNNs like ResNet-50. Meanwhile, the training time for DNNs is getting faster. Some networks could be fully trained in several hours. The network training time is much faster than the optimization time of the learning-to-compile approach. This makes the issue of reducing the compilation and optimization time of inference models even more critical. In addition, with the rapid development of DNNs, various DNN architectures have appeared in a short time. They need to be quickly deployed on different kinds of hardware platform. The lengthy process of optimization may hinder the development of DNNs, even questioned the practicability of the learning-to-compile approach.

Despite the efficacy of the learning-to-compile approach in generating highly optimized code for deep neural network (DNN) operators, a significant challenge persists: the protracted duration of the optimization process. For example, the optimization times for tools such as AutoTVM or AutoTune can extend to dozens of hours for some DNNs like ResNet-50. Currently, advances in DNN training methodologies have markedly reduced training durations, and some networks are fully trained in just a few hours. This discrepancy underscores the pressing need to accelerate the compilation and optimization times for inference models, rendering it a pivotal concern in the field. Furthermore, the rapid evolution of DNN architectures, coupled with the need to deploy them quickly on various hardware platforms, exacerbates the situation. The prolonged optimization timelines not only hinder the progress of DNN development, but also cast doubts on the practicality of the learning-to-compile strategy in keeping pace with the fast-evolving demands of deep learning technology.

To enhance the applicability of the learning-to-compile approach and address its inherent optimization time challenges, we present a novel mechanism named Roofline for Fast AutoTune (ROFT) . This innovative approach is designed to substantially accelerate the tuning process while maintaining, or even improving, the performance of DNNs. Our empirical investigations demonstrate that ROFT is capable of achieving optimization outcomes that are comparable to or surpass those obtained with established tools such as AutoTVM and AutoTune. In particular, it achieves these results in a fraction of the time, which presents a significant advancement for typical DNNs and operators. This efficiency improvement not only underscores ROFT’s potential to revolutionize the optimization landscape but also validates its contribution towards mitigating the critical bottleneck of prolonged optimization periods in the deployment and development of DNNs.

ROFT introduces a rule-based cost model alongside a flexible search algorithm, integrating the benefits of static analysis efficiency with the dynamic optimization capabilities of Learning-to-Compile approaches. This work proposes an advanced cost model informed by the Roofline Model^11^, enhanced with finer microarchitectural insights through the integration of scheduling parameters in calculating the arithmetic intensity. The model emphasizes the intensity of the arithmetic and execution concurrency, which are essential for microarchitecture-specific platforms. Moreover, we delineate the foundational principles of our rule-based cost model, designed for straightforward adaptation across diverse hardware environments, including GPUs and NPUs. The innovative cost model underpins our versatile search algorithm, which excels both as an independent performance optimization tool and as a preliminary filter to augment other automatic tuning systems. ROFT significantly narrows the search space for the configuration, simultaneously increasing the caliber of potential configurations. Consequently, ROFT adeptly identifies superior scheduling configurations on various target hardware platforms. This paper delineates the following significant contributions.

- We design and implement a roofline-like performance cost model, the so-called ROFT model, introducing the schedule parameters (knobs) into the expression of arithmetic intensity. The cost model is simple and effective for different target hardware platforms such as GPUs and NPUs. The ROFT model predicts the performance of operators based on the schedule parameters and microarchitecture features and significantly reduces the size of the search space.
- We propose a novel and flexible two-stage search algorithm, the so-called ROFT search algorithm. The ROFT search algorithm uses the aforementioned ROFT cost model for preliminary screening. Subsequently, it searches for the optimal configuration or optionally combines with existing machine learning algorithms, thus enhancing the performance of both the tuning process and operators.

The remainder of this paper is organized as follows. “Related work” section briefly covers related work on auto-tuning and the roofline model. “ROFT model” section presents the ROFT model that we proposed and provides a detailed exposition of the ROFT cost model and the ROFT search algorithm. “Performance evaluation” section discusses and analyzes the results of the experiment. Finally, in “Conclusion” section we conclude our work and provide directions for future research.

## Related work

### Auto-tuning

Deep learning (DL) compilers could automatically generate low-level optimized code for DNN models. For instance, TVM leverages an optimized computation graph to perform operator-level optimizations, thus generating high-performance code, especially for operators characterized by static shapes. Embracing the principle of computation / schedule separation^12^, operators are delineated through a declarative tensor expression language, allowing the abstraction of execution specifics. With the use of schedule templates and knobs, which represent schedule parameters, AutoTVM automatically explores a large schedule space to find the best configuration and generate optimized low-level programs.

Research works on tuning acceleration are divided mainly into two different directions. The first focuses on the refinement of machine learning algorithms through the integration of statistical methods^13,14^. A quintessential implementation of this approach is MetaTune^15^ , which conceptualizes the codes of deep neural network operators (DNN) as structurally analogous graphs. Using a graph neural network (GNN) as a cost model, MetaTune adeptly predicts the optimization parameters for DNN operators during the compilation phase. Currently, Merouani et al.^16^ have developed a deep learning-based cost model dedicated to automating code optimization, which processes a hierarchical representation of high-level features of unoptimized code to forecast potential speedups. In parallel, Gao et al.^17^ have introduced an innovative evolutionary strategy, augmented with a specialized mutation operation, to navigate search spaces for optimal scheduling parameters. Another notable contribution is CHAMELEON^18^, designed by the University of California for AutoTVM, which improves the conventional XGBoost + SA (Simulated Annealing) search methodology with a combination of Reinforcement Learning (RL) and Adaptive Sampling. Ahn et al.^19^ from the University of California innovatively proposed a method named Glimpse that mathematically embeds hardware specifications into the search process and combines it with meta-learning, significantly accelerating the search speed. Li et al.^20^ have proposed AdaTune, a novel tuning methodology that uses a random forest as a surrogate model and uses the coefficient of variation (CV) to accelerate the search process. Amazon Web Services does not aim to improve search algorithms, but rather enlarges the data set by sharing schedule configurations to improve AutoTVM’s search efficiency^21^. This method requires a large number of profiled schedule logs to cover various operators and improve the accuracy of the cost model. Unity^22^ introduces the concept of parallel computation graphs and uses algebraic optimization. It efficiently searches for optimal scheduling strategies for different hardware platforms through a multi-level search algorithm, effectively improving the performance of the model. DOPpler, which is proposed by Borowiec et al.^23^, accelerates the optimization of tensor programs by executing candidate programs in parallel between CPU hosts and GPU devices, reducing the auto-tuning time without sacrificing performance. The method proposed by Yang et al.^24^ integrates the RISC-V Packed extension into TVM, to facilitate efficient deep learning computations on edge devices with enhanced performance and minimal accuracy loss. Li et al.^25^ proposed an auto-tuning framework named FamilySeer, which improves the efficiency of the generation of tensor programs by exploiting the similarities among subgraphs.

The second involves using static analysis. For example, Amazon Web Services and the University of Utah propose Tuna^26^ , which optimizes deep learning programs by performing static analysis on the relative performance of tensor operations. Tuna parses intermediate representations of high-level programs and low-level assembly code on TVM. Although Tuna relies solely on static analysis, the cost model is intricate and detailed. For certain operators, Tuna achieves performance optimization similar to that of AutoTVM. However, for other DNN operators, it still exhibits a performance gap compared to AutoTVM.

Meanwhile, new automatic compilation tools continue to emerge, with auto-scheduling gradually becoming the focus of research. Auto-scheduling refers to a scenario where users only need to provide the compute interface, and the compilers generate efficient execution code automatically. For example, Ansor^27^ is an auto-scheduling module in TVM. Essentially, it automatically generates schedule templates with various knobs. However, the process of identifying the optimal configuration from a vast pool of candidates remains a formidable challenge, a gap precisely addressed by our ROFT methodology.

Compared with other methods, ROFT combines the efficiency advantage of static analysis with the performance advantage of auto-tuning. It employs a simple and direct rule-based cost model to pre-filter the search space and select promising configurations, enabling targeted searches within a limited space to identify the best configuration.

### Roofline model

The efficiency of inference execution for deep learning algorithm models is significantly dictated by the underlying hardware performance. A nuanced understanding and integration of hardware-specific characteristics are paramount to optimizing these models’ inference time. By tailoring computational strategies to align with the unique attributes of the hardware platform, it becomes feasible to not only enhance model efficiency but also to leverage the full potential of the hardware, thereby achieving optimal performance outcomes.

The Roofline Performance Model^11^, which is abbreviated as the Roofline Model, can be used to analyze the theoretical computational performance limit that a model can achieve on a specific computing platform. Due to factors such as experimental conditions, actual performance test results typically fall short of those provided by the Roofline Model. Hence, the roofline model has been used for performance analysis and optimization of both NVIDIA and AMD GPUs^11,28^. Recently, scholars have expanded its application to analyze and optimize the performance of AI operators^29^.

The Roofline Model can be used by evaluating the theoretical maximum computational performance achievable by a model on a given computing platform. It offers a ceiling for computational efficiency, delineating the optimum performance boundary. However, due to a variety of factors including experimental setups and operational constraints, actual performance metrics often do not reach the theoretical peaks suggested by the Roofline Model. This disparity has led to the widespread application of the model in performance analysis and optimization efforts for computing architectures, including NVIDIA and AMD GPUs^11,28^ . The utility of the Roofline Model has recently been expanded to include the analysis and enhancement of AI operator performance^29^, reflecting its adaptability and relevance in the evolving landscape of computational technologies.

The upper limit of theoretical computational intensity, denoted as ${I\_{{max}}}$, describes the maximum number of computations that can be performed per unit of memory exchange on this computing platform. The higher the computational intensity, the higher the memory usage efficiency. ${I\_{{max}}}$ can be represented by Formula (1):

1 $$\begin{aligned} {I\_{{max}}} = \frac{\pi }{\beta } \end{aligned}$$

In this context, $\pi$ represents the computational power, also known as the upper limit of the performance of the computing platform, which denotes the number of floating point operations that the platform can perform per second at full capacity. The unit is Flops. $\beta$ signifies the bandwidth, which is the upper limit of the computing platform bandwidth, indicating the maximum amount of memory exchange that can be done per second at full capacity. The unit is Bytes/s.

**Fig. 1:**
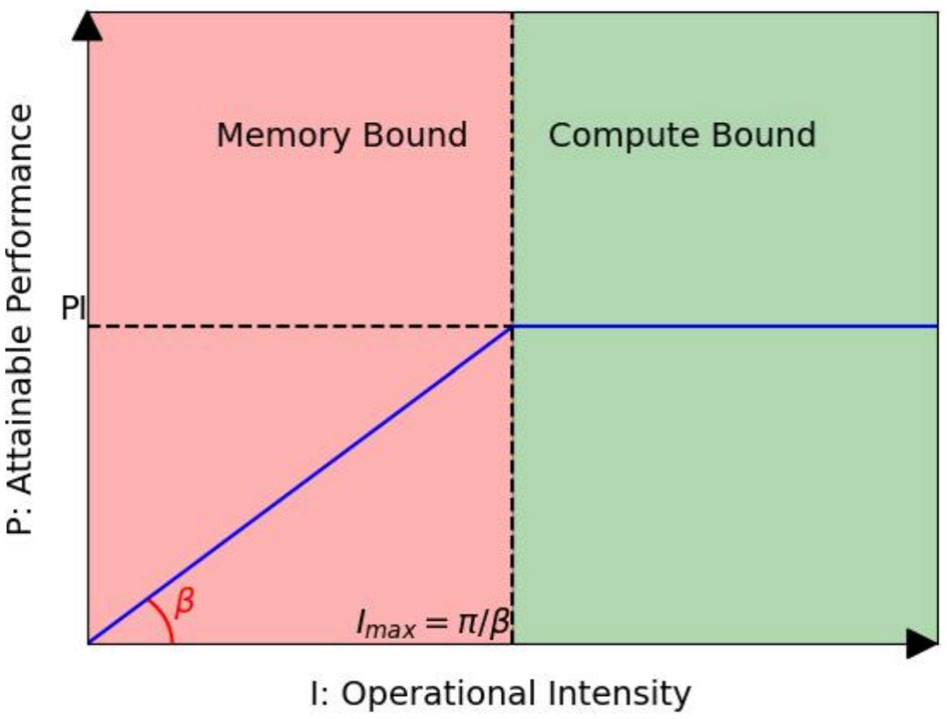
Roofline model.

The Roofline Model is illustrated in Fig. 1, with the horizontal axis representing computational intensity *I* and the vertical axis representing theoretical peak performance *P*. As depicted, computational power determines the “roof” height (green line segment), while bandwidth determines the slope of the ”eaves” (red line segment).

According to the Roofline Model, two bottleneck regions are delineated in Formula (2):

2 $$\begin{aligned} P = {\left\{ \begin{array}{ll} \beta \cdot I & \text {when } I < I_{\text {max}} \quad \text {Memory Bound} \\ \pi & \text {when } I \ge I_{\text {max}} \quad \text {Compute Bound} \end{array}\right. } \end{aligned}$$

Compute Bound Region: irrespective of the computational intensity *I* of the model, its theoretical performance *P* can at most equal the computational power $\pi$ of the computing platform. When the computational intensity *I* of the model exceeds the upper limit of the computational power ${I\_{{max}}}$ of the computing platform, the model operates in a Compute Bound state. In this state, the theoretical performance *P* of the model is constrained by the computational power $\pi$ of the platform and cannot scale linearly with the computational intensity *I*. However, this is not necessarily unfavorable, as it indicates the model is utilizing 100% of the computational power available on the platform. It is evident that as the computational power $\pi$ of the platform increases, the theoretical performance *P* of the model after entering the compute bound region also increases.

Memory-bound region: when the computational intensity *I* of the model is less than the upper limit of computational power ${I\_{{max}}}$ of the computing platform, the model resides in the “eaves” interval. Consequently, the theoretical performance *P* of the model is completely determined by the bandwidth $\beta$ (the slope of the eaves) of the computing platform and the computational intensity *I* of the model. Hence, this state is termed Memory Bound. It is evident that within the memory bound region, the larger the bandwidth $\beta$ of the computing platform (the steeper the eaves), or the greater the computational intensity *I* of the model, the model’s theoretical performance *P* can exhibit linear growth.

## ROFT model

### Overview

**Fig. 2:**
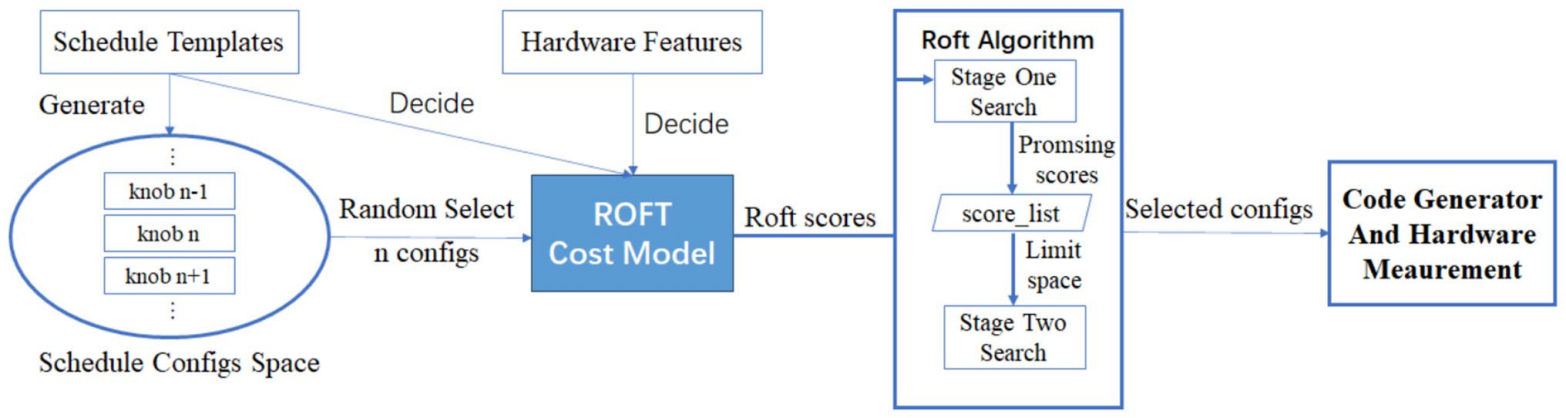
ROFT overall design.

Figure 2 presents the overall design of ROFT, including a rule-based cost model and a two-stage search algorithm.

Based on the concept of compute/schedule separation, TVM requires developers to write schedule optimization templates after describing the computation. Knobs can be defined in the schedule templates to form a vast schedule space by combining their values. The ultimate goal of automatic tuning is to find configurations of knobs that yield better performance. The ROFT cost model automatically calculates ROFT scores for all configurations, which can be used to obtain an ordered set of configurations with performance ranging roughly from high to low based on their ROFT scores, arranged from high to low.

The ROFT search algorithm consists of two stages. The first stage aims to identify a set of promising ROFT scores that represent better operator performance. It involves obtaining *n* configurations with different ROFT scores, from which only one configuration is selected for each distinct ROFT score to be measured on the specified hardware. The measurements are conducted in descending order of ROFT scores as they roughly correspond to the actual performance of the configurations. To optimize search time, an early stop mechanism is employed in both stages of the search to stop the search if the performance of the top configurations continues to decrease.

Upon completion of the first stage, a score list is generated that records ROFT scores and their corresponding schedule configurations. The second stage then re-evaluates the schedule space according to the score list, searching for different configurations with the same ROFT scores as candidates and executing them on the specified hardware. This process can be repeated to achieve the best performance in a shorter period of time. Ultimately, ROFT will find the configuration with the best performance.

### Cost model

The ROFT cost model, which is a roofline-like cost model, can be automatically calculated from schedule templates. We will present the fundamental principles of the cost model as well as implementations on NVidia GPUs and Huawei Ascend NPUs for typical operators such as convolutions.

#### Design principle

The Roofline model proposes a quantitative analysis method using the arithmetic intensity^30^. It can roughly predict the upper performance limits for DNN operators. However, these predicted results are likely to be preliminary and inaccurate without considering the internal details of the programs and microarchitectures. As such, it cannot be used directly as a cost model.

First, computing tasks are typically deployed on different microprocessor cores and executed concurrently on multicore architectures. As a result, a cost model should take into account the impact of concurrency. Secondly, when focusing on the execution of kernel functions, the calculation method of arithmetic intensity should consider the details of schedule optimizations in operators at a lower level. This means that more schedule configurations should be considered when calculating the arithmetic intensity, not only taking into account the calculation quantities and rates of outside I/O but also different data tiles and inside I/O. Lastly, other vital parameters of the knobs should also be considered to represent the calculation modes.

In light of these considerations, we present the abstract formula for the ROFT cost model, which includes the kernel’s arithmetic intensity *I_kernel* and the *Concurrency*, as Formula (3):

3 $$\begin{aligned} Score = I\_kernel \times Concurrency \end{aligned}$$

Similarly, the relationship between the kernel’s arithmetic intensity *I_kernel* and the kernel’s arithmetic amount *A_kernel* and the kernel’s memory traffic *T_kernel* can be expressed as Formula (4):

4 $$\begin{aligned} I\_kernel = A\_kernel \div T\_kernel \end{aligned}$$

Then we can express *A_kernel*, *T_kernel* and *Concurrency* with different *knobs* in schedule templates. According to the computing process, they can be easily implemented. The abstract presentations are shown as formulas (5),  (6), and  (7):

5 $$\begin{aligned} A\_kernel&= f_a(knob0,knob1,knob2...)\end{aligned}$$

6 $$\begin{aligned} T\_kernel&= f_t(knob0,knob1,knob2...)\end{aligned}$$

7 $$\begin{aligned} Concurrency&= f_c(knob0,knob1,knob2...) \end{aligned}$$

The different schedule knobs represent a variety of schedule policies, such as the allocation of storage buffers, the usage times of computing units, and the different parallelization modes. Although it is not possible to consider all factors, adding more knobs to the formulas is still helpful for low-level models because the arithmetic intensity of different schedule configurations presents the relationship between I/O and arithmetic amounts, which are critical performance indicators. Therefore, we can use the ROFT cost model design principle to evaluate different schedule configurations with more representative performance scores.

In addition, various schedule templates may have diverse cost models due to different data flows and calculation patterns. However, modifying the functions for calculating the arithmetic intensity and concurrency is all that is required. Thus, we can easily incorporate cost models for various operators and microarchitectures.

#### Cost model for NVidia GPUs

We will use convolution operators as examples to demonstrate how to implement the ROFT cost model for a specific microarchitecture. Convolution operators are critical in modern deep learning models, particularly CNN models. We will use the Conv2D schedule template for AutoTVM and NVidia GPUs in the following.

Table 1 lists the optimization knobs for Conv2D in the AutoTVM schedule template for NVidia GPUs. The major goals of the optimized code include: (1) maximizing data reuse; (2) making extensive use of shared memory; (3) minimizing bank conflicts, etc. These knobs attempt to optimize the operator at multiple execution levels, such as tiling (e.g. $tile\_f$ and other knobs) and unrolling (e.g. $auto\_unroll\_max\_step$ and other knobs). Together, all these knobs define a vast optimization design space, which could be on the order of $10^9$. With the eight primary knobs and the input and output parameters of the operator listed in Table 1, we can implement the ROFT cost model on GPU architectures for the Conv2D operator.

**Table 1:** Primary Conv2D optimization knobs in the schedule template of AutoTVM for NVidia GPUs.

Knobs	Definition
tile_f,tile_y,tile_x	Data and weight factors for tiling and binding
tile_rc,tile_ry,tile_rx	Channels, height and width of filters for tiling reduction axis
auto_unroll_max_step	Threshold of unrolling steps of loops
unroll_explicit	Explicitly unrolling loops

As per the aforementioned design principle of the ROFT cost model, the arithmetic amount *A_kernel* of the operator will be calculated in the first step, as Formula (8):

8 $$\begin{aligned} A\_kernel&= 2\times tile\_f[3]\times tile\_y[3]\times tile\_x[3]\nonumber \\&\times tile\_f[1]\times tile\_y[1]\times tile\_x[1]\nonumber \\&\times tile\_rc[1]\times tile\_ry[1]\times tile\_rx[1]\nonumber \\&\times tile\_rc[0]\times tile\_ry[0]\times tile\_rx[0] \end{aligned}$$

The *tile_f*, *tile_y*, and *tile_x* are data and weight factors used for tiling and binding in the convolution. The variables *tile_rc*, *tile_ry*, and *tile_rx* are used to til the reduction axis of the filter channel, height, and width, respectively. When calculating the kernel’s memory traffic *T_kernel*, three different I/O overheads will be calculated, including the left operand I/O *data_io*, the right operand I/O *weights_io*, and the output I/O *out_io*. The abstract presentations are shown as formulas (9),  (10), and  (11):

9 $$\begin{aligned} data\_io&= data\_shape[2]\times weight\_shape[2] \nonumber \\&\div (tile\_f[2] \times tile\_y[2]\times tile\_x[2] \nonumber \\&\times tile\_y[0]\times tile\_x[0]) \end{aligned}$$

10 $$\begin{aligned} weights\_io&= weight\_shape[2]\div (tile\_f[2] \nonumber \\&\times tile\_y[2] \times tile\_x[2]\times tile\_f[0]) \end{aligned}$$

11 $$\begin{aligned} out\_io&= tile\_f[3]\times tile\_y[3]\times tile\_x[3] \nonumber \\&\times tile\_f[1]\times tile\_y[1]\times tile\_x[1] \end{aligned}$$

And then, the arithmetic density of a single kernel is shown as Formula (12):

12 $$\begin{aligned} I\_kernel&= A\_kernel \div T\_kernel\nonumber \\&= A\_kernel\div (data\_io\nonumber \\&+ kernel\_io + out\_io) \end{aligned}$$

Next, * concurrency* could be calculated considering two main factors, ie the number of blocks and the number of warps in a single block, which mainly impact the concurrency of kernel functions on GPUs. For some NVidia GPUs, the number of blocks is more than three times the number of streaming processors (SPs), which leads to better performance. So, we define the parameter $\alpha$ as Formula (13):

13 $$\begin{aligned} \alpha = \left\{ \begin{array}{ll} 1 & \text {if } block > SPs\times 3 \\ block\div (SPs\times 3) & \text {if } block \le SPs\times 3 \end{array} \right. \end{aligned}$$

We incorporate a variable $\beta$ with $\alpha$ to adjust the parameter *Concurrency* for considering how many warps are in a single block. It equals 0.5, if the number of warps in a block is 1, otherwise it equals 1. We define the parameter $\beta$ as the formula (14):

14 $$\begin{aligned} \beta = \left\{ \begin{array}{ll} 1 & \text {if } 1<warps \\ 0.5 & \text {if } warps \le 1 \end{array} \right. \end{aligned}$$

Finally, the *Concurrency* is quantified as Formula (15) for simplicity:

15 $$\begin{aligned} Concurrency=\alpha \times \beta \end{aligned}$$

Up until now, the ROFT cost model on NVidia GPUs has been completely implemented.

#### Cost model for Huawei Ascend310 NPUs

For comparison purposes, we also implemented the ROFT cost model for the Conv2D operator using the Huawei Tensor Boost Engine (TBE) on the Huawei Ascend310 NPU.

Unlike conventional CPUs and GPUs, which are used for general-purpose computing, or some ASIC architectures designed for specific algorithms, the DaVinci architecture of the Huawei Ascend310 NPU is intended for common applications and algorithms in specific fields, such as deep learning. Therefore, it is often referred to as a domain-specific architecture (DSA).

**Fig. 3:**
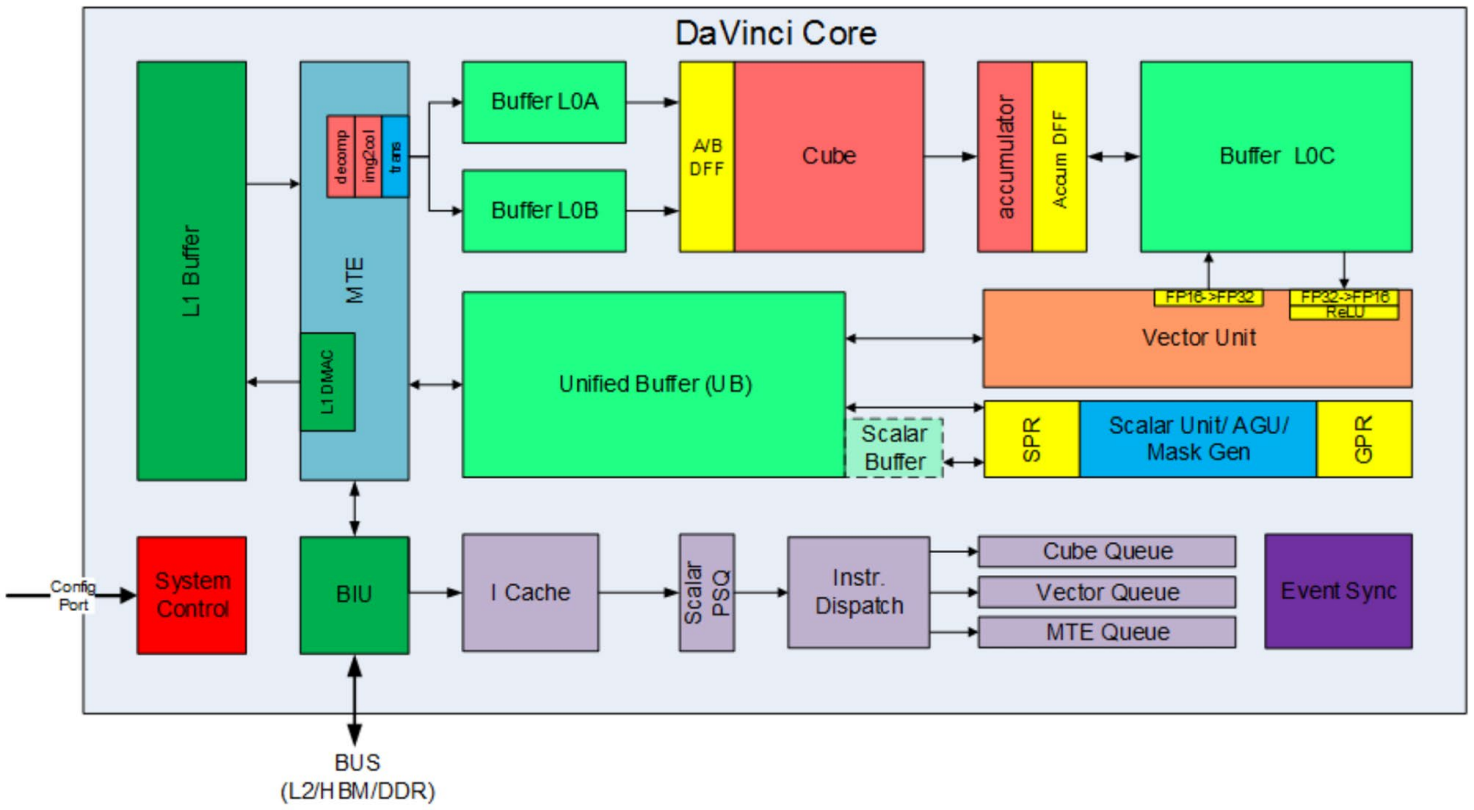
DaVinci architecture^31^.

As the computing core of the Ascend310 NPU, AICore can be considered a simplified architecture of modern microprocessors, as shown in Fig. 3. It consists of three basic computing units: the cube unit, the vector unit, and the scalar unit. These three compute units have different parts to play with, forming three independent pipelines that fit together under the unified scheduling of system software to achieve optimized compute efficiency.

Because of the significant difference in computing efficiency among the compute units, the ROFT cost model will mainly focus on the cube unit, which is much more powerful when performing matrix computation compared to others. Each time the cube is executed, a multiplication of the matrix $16 \times 16$ will be completed at fp16. For formulas like $C = A \times B$ where *A* from buffer L0A and *B* from buffer L0B, the matrix multiplication and the intermediate results will be stored in buffer L0C.

In a schedule template for TBE, the execution process of Conv2D can be described as follows: First, the input data and weights are sent from global memory to buffer L1 and then from buffer L1 to buffer L0, or from global memory to buffer L0 directly, by MTE (multiple memory transfer engines). Some knobs in the schedule template define the data flow. Secondly, the left operand is reshaped by the MTE Img2Col routine to maximize the usage of the cube unit. Then, when the left operand in buffer L0A and the right operand in buffer L0B are both ready, the cube unit performs the $16 \times 16$ matrix multiplication. If the input matrix is larger than the input size of the cube unit, the cube unit tiles the larger matrix to fit its input size and repeats this process. Finally, when the computation is finished, the output results are sent to the unified buffer and later to the global memory.

Table 2 lists knobs of the TBE Conv2D schedule template, which aim to achieve objectives such as: (1) maximizing the data reuse in buffers; (2) minimizing the synchronization overhead; (3) maximizing the usage of the cube unit, etc. These knobs optimize multiple levels of execution, e.g., different buffer sizes (Buffer AL1, BL1, etc.), the flag of using double buffers and the parallel binding (assignment of tasks on different AICores), etc.

**Table 2:** Knobs of the TBE Conv2D schedule template.

Knobs	Definition
AL1_shape,BL1_shape	Allocate buffer L1 to data, weights
AL0_matrix,BL0_matrix, CL0_matrix	Allocate buffer L0 to data, weights, outputs
AUB_shape,BUB_shape, CUB_shape	Allocate unified buffer to data, weights, outputs
(AL1,BL1,AL0,BL0,CL0, AUB,BUB,CUB)_pbuffer	Decide whether to set double buffer at given every on-chip buffers
block_dim	Conv’s data factors for tiling and binding on each AICore
(A,B)_overhead_opt_flag, n_bef_group_flag, n_bef_batch_flag	Some schedule adjustments that are difficult to estimate

As shown in Table 3, some knobs describe the shapes of the data, e.g. $$(N_{a},H_{a},W_{a}$$, $$C_{a})$$ for the shape of input data, $$(N_{b},H_{b},W_{b}$$, $$C_{b})$$ for the shape of weights and $$(N_{o},H_{o},W_{o},C_{o})$$ for the shape of output.

**Table 3:** Shapes of data in different buffers.

Location	DATA_SHAPE	Size
gm	$(N_{a},H_{a},W_{a},C_{a})$	$S_{gm}\_a$
L1	$(N_{a},H_{a} \times W_{a} \div 16,C_{a},H_{b},W_{b},16)$	$S_{L1}\_a$
L0	$(N_{a},H_{a} \times W_{a} \div 16\div 16,H_{b}\times W_{b},16,16)$	$S_{L0}\_a$

Given the knobs and input/output parameters of an operator, we can implement the ROFT cost model on the Ascend310 NPU (with two AICores) as below.

Firstly, the calculation amount *A_kernel* of the operator is defined as formula (16):

16 $$\begin{aligned} A\_kernel&= N_{o}\times H_{o}\times W_{o}\times C_{o} \nonumber \\&\times H_{b}\times W_{b}\times C_{b}\div block\_dim \end{aligned}$$

Secondly, for computing the kernel’s memory traffic *A_kernel*, we should take into account multiple-layer on-chip buffers and different I/Os, including the left operand I/O *data_io*, the right operand I/O *weights_io* and the output I/O *out_io*, etc. We use *S* to represent the sizes needed in different buffers. Based on knobs in Table 3, the memory traffic *T_kernel* will be divided into three parts, as Formulas (17),  (18), and  (19):

17 $$\begin{aligned} data\_io&= S_{L1}\_a \div AL1\_shape + S_{L0}\_a \div AL0\_matrix\end{aligned}$$

18 $$\begin{aligned} weights\_io&= S_{L1}\_b \div BL1\_shape + S_{L0}\_b \div BL0\_matrix\end{aligned}$$

19 $$\begin{aligned} out\_io&= S_{L0}\_c \div CL0\_matrix + S_{UB}\_c \div CUB\_shape \end{aligned}$$

Thirdly, we can get the arithmetic density of a single kernel, as Formula (20):

20 $$\begin{aligned} I\_kernel&= A\_kernel \div T\_kernel\nonumber \\&= A\_kernel\div (data\_io\nonumber \\&+ kernel\_io + out\_io) \end{aligned}$$

Finally, the * currency* of multiple AICores should be taken into account. Ascend310 has two AICores. The knob named $block\_dim$ controls their working. In addition, the use of double buffers influences instruction-level parallelism. Considering both of them, we define *Concurrency* as Formula (21):

21 $$\begin{aligned} Concurrency=&block\_dim\times \nonumber AL1\_pbuffer\times \nonumber \\&BL1\_pbuffer\times AL0\_pbuffer\times \nonumber \\&BL0\_pbuffer\times CL0\_pbuffer\times \nonumber \\&CUB\_pbuffer \end{aligned}$$

Up until now, the ROFT cost model for the Ascend310 NPU has been completely implemented.

### Search algorithm

The ROFT cost model predicts the performance of the operators in a simple and fast way. However, it could not be used to choose the best configuration without runtime evaluations. The ROFT search algorithm is proposed to seek better performance in practice. It can be divided into two stages. Algorithms 1 and 2 show the specific execution stages of the ROFT search algorithm.

As shown in Algorithm 1, the first stage aims to build mappings between the ROFT cost model and the actual performance. This stage will obtain a batch of random configurations and compute their ROFT scores using the cost model. After ranking them according to their scores from high to low, we select only one configuration from those with the same ROFT score and measure it on the target device. In order to avoid some unnecessary measurements, an early stop mechanism, which will stop the evaluation process if the performance of configurations is continuously worse than the current average performance, will be applied to this process. After the execution of the first stage, we will get a $score\_list$ that includes the top ROFT scores with their corresponding actual performances.

**Algorithm 1:**
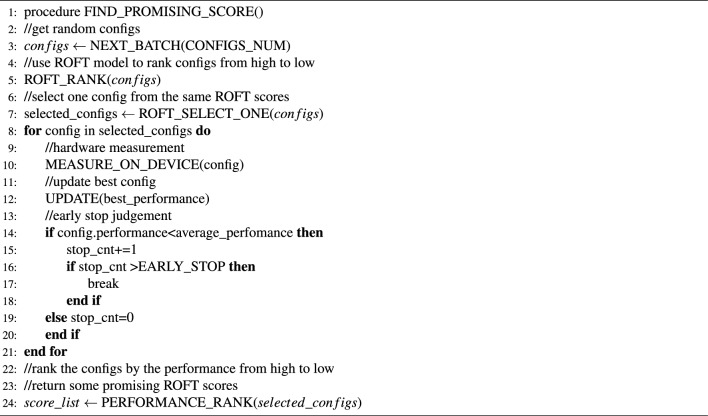
ROFT search algorithm Stage I

**Algorithm 2:**
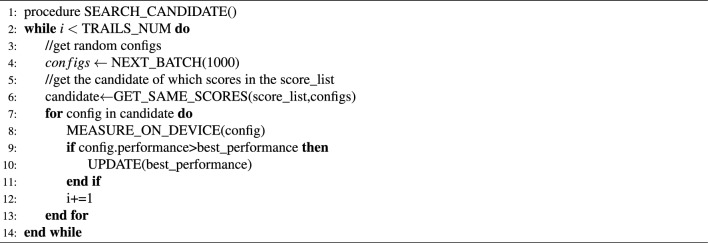
ROFT search algorithm Stage II

As shown in Algorithm 2, the second stage aims to reduce the schedule space by using the $score\_list$ generated in the first stage along with its corresponding real performance. This stage will select a batch of random configurations and compute their ROFT scores using the cost model first. Then, only the configurations with the same ROFT scores in $score\_list$ will be compiled and executed on the target device. Because it is difficult to quantify all the knobs precisely in our cost model, some configurations with quite different actual performance could have the same scores. We will choose only one configuration for every score, while the total number of configurations chosen is less than 500 for most operators on both GPUs and Huawei Ascend NPUs. Because the schedule space is significantly reduced compared to the previous one, which was as large as $10^{9}$ on GPUs, it is small enough to use a simple and efficient random search to find the best performance. As long as the configurations are found, they will be measured on the actual device. This stage could be processed repeatedly until the execution time reaches $TRAILS\_NUM$. Ultimately, we may obtain an efficient operator configuration using the ROFT search algorithm.

Using the two-stage ROFT search algorithm, we found that some so-called hyperparameters about the search process will also affect the final search efficiency and performance. Table 4 lists two hyperparameters and their definitions. Practical experience indicates that the CONFIGS_NUM should be set to larger. That means that the more ROFT scores can be covered, the better performance could be achieved. However, it is time-consuming if the number is too large due to the subsequent sort algorithm. For example, we usually set CONFIGS_NUM=5000, EARLY_STOP=20, and TRAILS_NUM=100 for the balance of search time and final performance.

**Table 4:** Hyper parameters in the ROFT search algorithm.

Hyper-parameters	Definition
CONFIGS_NUM	The size of the batch selected from the schedule space
EARLY_STOP	Threshold number of early stop
TRAILS_NUM	The number of configs will be executed on real device in Stage 2

## Performance evaluation

In this section, we present the results of the performance evaluation of the ROFT model on NVidia GPUs ( i.e., the GTX 1060, Tesla T4, and Tesla V100) and the Huawei Ascend310 NPU. In this experiment, we used two Intel Cascade Lake 6278 CPUs (2.6GHz) with 8GB of memory on the host machine as well. We implemented the ROFT cost model and search algorithm using TVM (v0.8.0 release) for NVidia GPUs and TBE (CANN20.0 alpha) for Huawei Ascend NPUs.

We evaluated the ROFT model in the following three aspects: the prediction ability of the cost model, the effectiveness of the two-stage search algorithm, and the optimized performance of some typical convolution operators and end-to-end DNNs. We compare the ROFT model with AutoTVM on GPUs and TBE AutoTune on Ascend NPUs. AutoTVM uses XGBoost or GA as the performance model and Simulated Annealing (SA) as the optimizer. The AutoTune model of TBE utilizes GA as a performance model.

### Prediction ability of ROFT cost model

In this subsection, we use one layer of the Conv2D operator in VGG-16^32^ to demonstrate the prediction ability of the ROFT cost model. The specific parameters of the Conv2D operator are shown in Table 5.

**Fig. 4:**
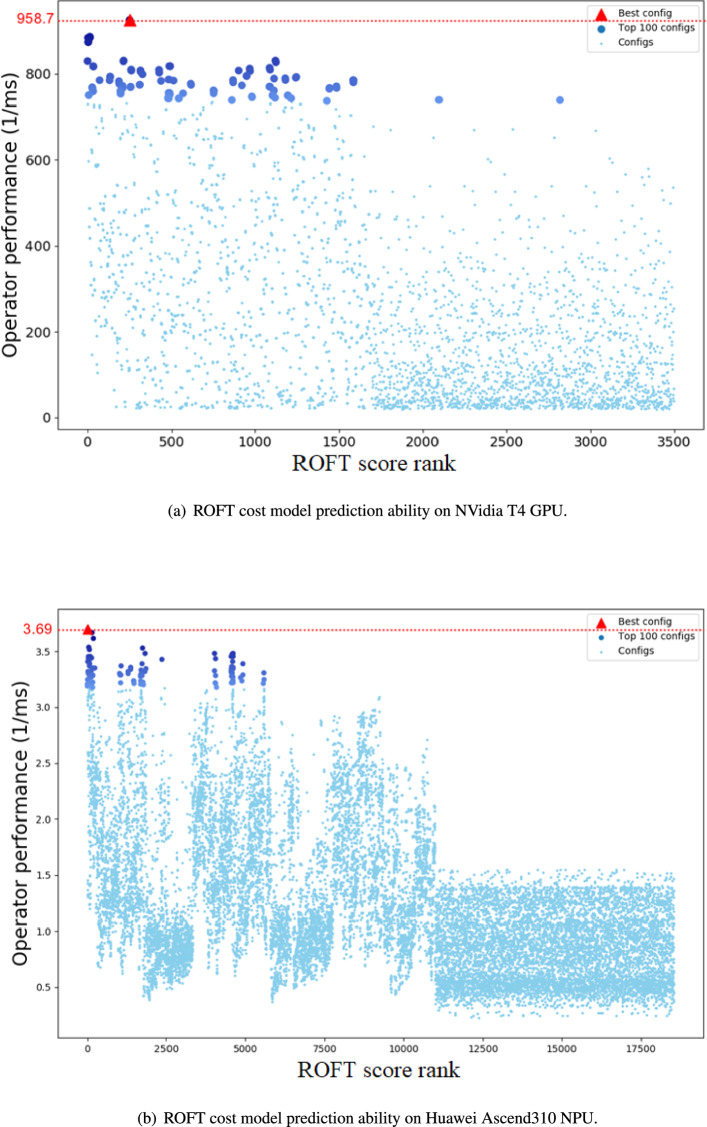
ROFT cost model prediction ability on GPUs and NPUs..

We randomly selected some schedule configurations from the AutoTVM and TBE schedule space. Due to the enormous size of the schedule space, measuring all configurations would be extremely time-consuming. Instead, we present a subset of random configurations to provide a glimpse of the entire space. Figure 4 shows the prediction ability of the ROFT cost model. In these figures, the X-axes represent the ranks ordered by ROFT score from high to low, while the Y-axes represent the actual performance expressed by the reciprocal of the operator execution time. The top 100 best configurations are marked in figures. The experimental results reveal that the ROFT cost model can appropriately evaluate performance. Upon examination of the cost model ranking, we observe that the better performing configurations are located within a range of higher ROFT scores. Moreover, configurations with poorer performance tend to cluster among the lower ROFT ranks.

**Table 5:** Parameters of the Conv2D operator.

Parameters	Values	Definitions
data_shape	(1,128,112,112)	Batch_size,in_channels, height and width
weight_shape	(128,128,3,3)	Out_channels, in_channels and kernel size
padding	(1,1)	Same padding
strides	(1,1)	Stride size

**Fig. 5:**
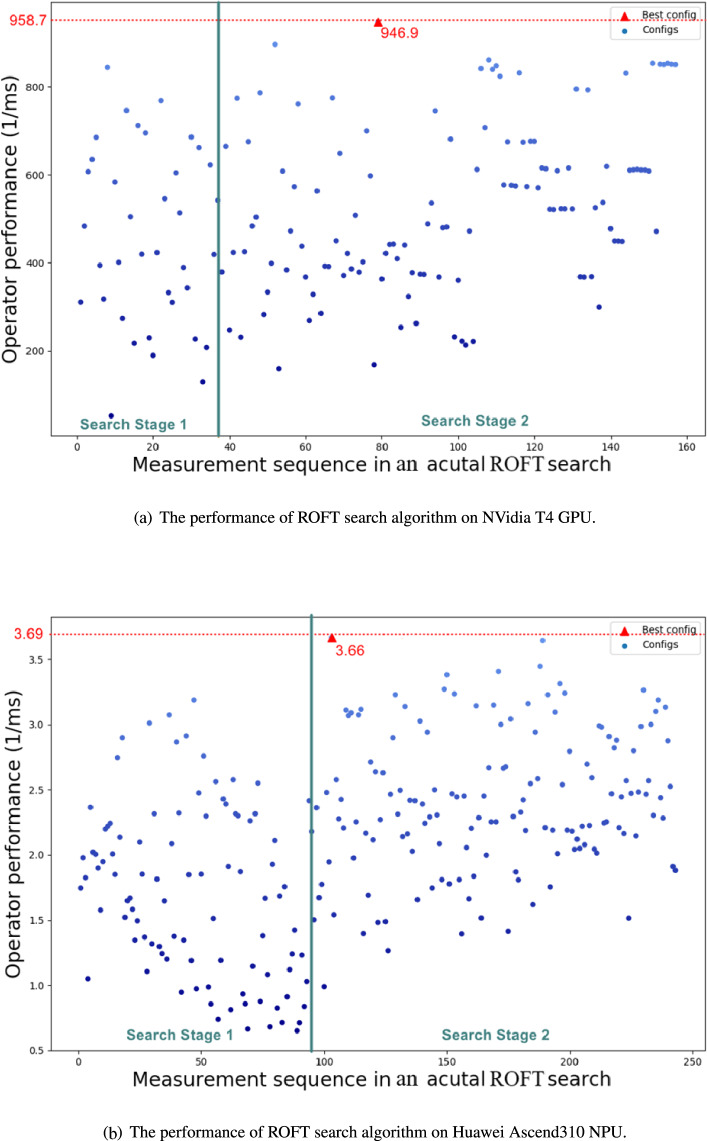
The performance of the two-stage ROFT search algorithm on GPUs and NPUs.

### Two-stage ROFT search algorithm

Although the ROFT cost model predicts performance with a certain level of credibility, as depicted in Fig. 4, it is not straightforward to rely solely on the cost model itself to choose the better configuration due to prediction errors. In practice, there can be an enormous number of configurations with the same ROFT score but with significant performance differences. This phenomenon is also illustrated in Fig. 4. Therefore, we employ a simple but effective two-stage ROFT search algorithm to identify superior configurations within the schedule space.

Figure 5 shows the performance of the two-stage ROFT search algorithm for Conv2D operators in VGG-16. Each data point in the figure represents a configuration selected by the ROFT search and measured on GPUs and NPUs. The X-axis represents the sequence of measurements for the configurations in the ROFT search process. For instance, the first evaluated configuration is marked as No. 1, and so on. The Y-axis represents the operator performance, which is expressed as the reciprocal of the operator execution time. In the first stage, there is significant variation in configuration performance to identify promising ROFT scores. In the second stage, we observe a general improvement in configuration performance compared to the first stage, and the best configuration is typically selected in the final stage.

Figures 4 and 5 represent the actual configuration performance within the overall schedule space and during the actual ROFT search process, respectively. By combining Figs. 4 and 5, it becomes apparent that the ROFT search algorithm effectively filters out most configurations with poorer performance. For example, configurations with worse performance can be defined as those below 200 $ms^{-1}$ on GPUs and 1.0 $ms^{-1}$ on NPUs. In Fig. 5, we observe that only a small number of configurations with worse performance are selected for compilation and execution on the target device, resulting in a significant reduction in tuning time. Furthermore, as illustrated in Fig. 4 for both microarchitectures, the performance of the top configurations is close to the actual best. The number of configurations surrounding the top performance, as selected by the ROFT search, is quite limited. This finding further highlights the stability of the ROFT search algorithm.

### Performance of operators and DNNs

**Fig. 6:**
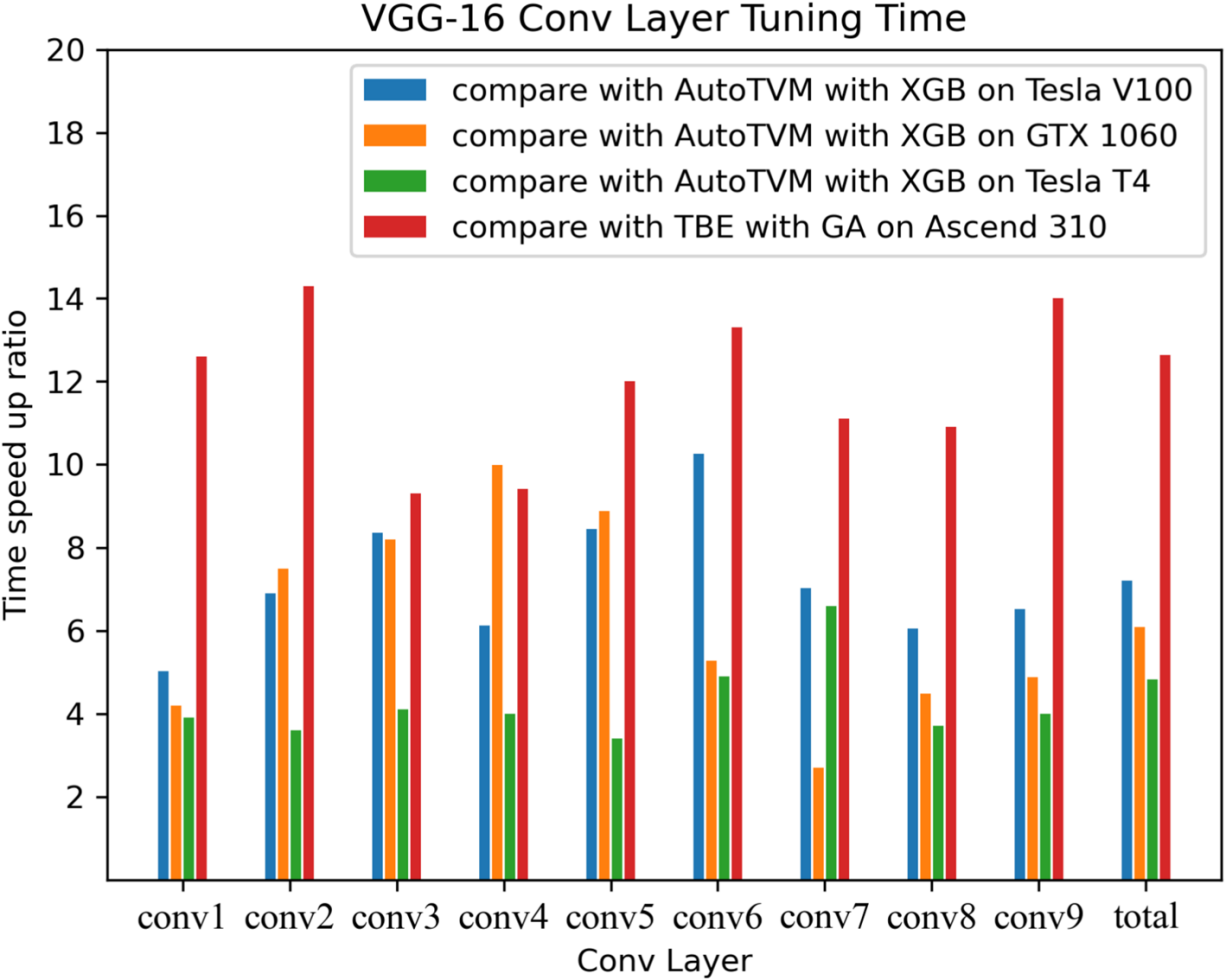
Tuning time speed-up of VGG-16 Conv2D operators on GPUs and NPUs.

**Fig. 7:**
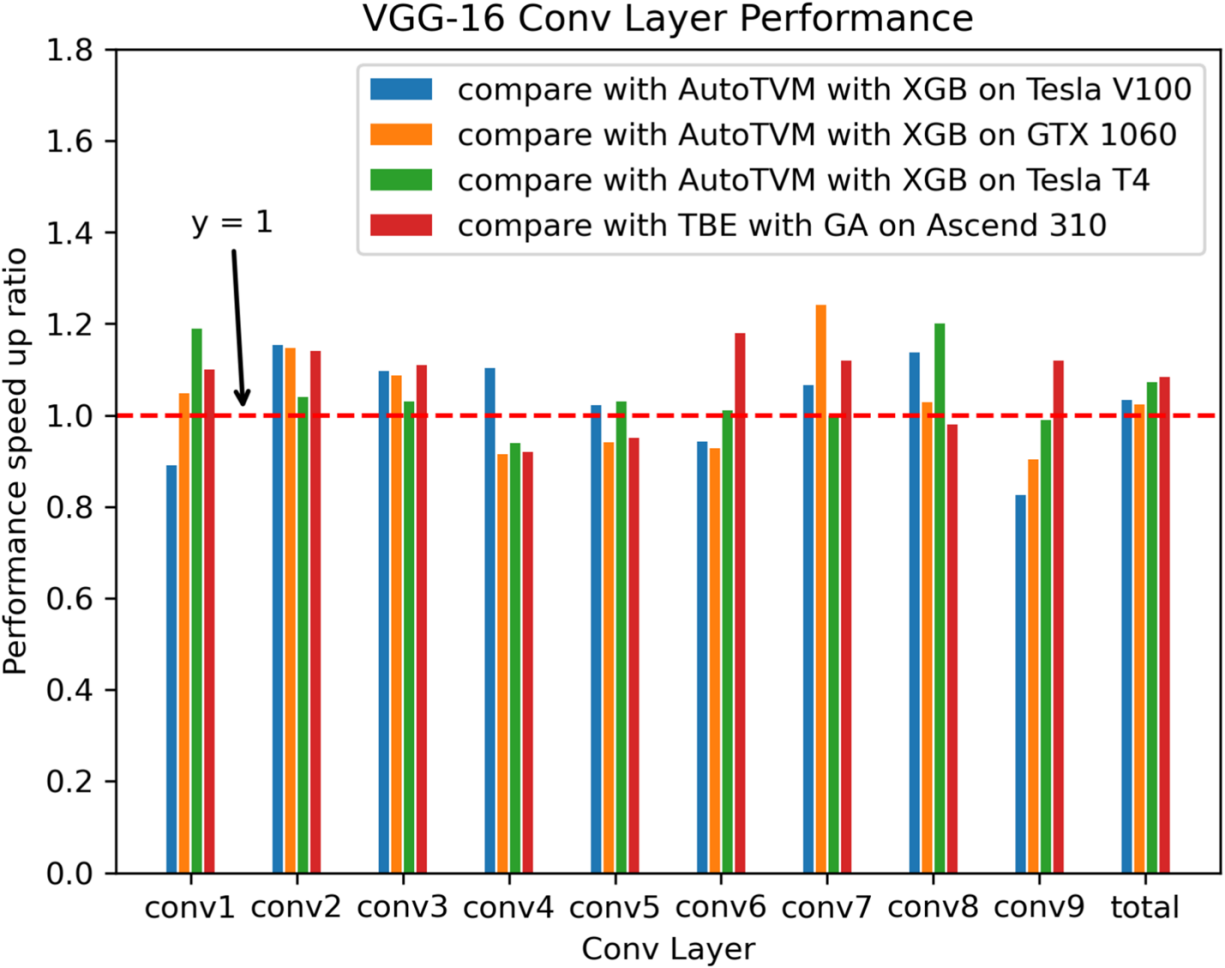
Performance speed-up of VGG-16 Conv2D operators on GPUs and NPUs.

We begin by evaluating the performance of optimized DNN operators using ROFT. In this experiment, we conducted comparative experiments using AutoTVM and TBE on different GPUs and NPUs. AutoTVM uses XGBoost as the performance model, while TBE utilizes GA as the performance model. The experimental results are shown in Fig. 6. As can be seen, on GPUs, the tuning time speed-up achieved by ROFT compared to AutoTVM ranges from a maximum of 10x on NVidia Tesla V100 to a minimum of 2.7x on NVidia GTX1060. Compared with TBE AutoTune on Huawei Ascend310 NPUs, ROFT-accelerated tuning time speed-up ranges from a maximum of 14.3x to a minimum of 9.3x. Furthermore, as shown in Fig. 7, most ROFT-optimized operators exhibit performance that is either better than or relatively close to baseline performance on GPUs and NPUs. Some operators get performance improvements of up to 20%. Furthermore, it can be observed that the use of ROFT leads to overall performance enhancements for the VGG-16 network compared to other methods. On GPUs, performance can be improved by up to 7.3%, and on NPUs, performance can be improved by up to 8.3%.

Finally, we present the end-to-end performance using a set of pre-trained DNN models. The selected models include popular deep learning applications for image classification and object detection, such as VGG-16, ResNet-50, ResNet-18, and the lightweight network MobileNet^32–34^. Due to the lower computational power of the GTX1060, we performed experiments using the relatively simpler network architecture of ResNet-18 on the GTX1060. Tables 6 and 7 respectively show the tuning time and performance of ROFT, AutoTVM, TBE, Chameleon and Glimpse. Compared to AutoTVM using XGBoost performance models, ROFT achieves an average speed-up ratio of approximately 6X on GTX1060 and Tesla V100, and approximately 4X on Tesla T4. Compared to AutoTVM using GA performance models, ROFT achieves an average speed-up ratio of approximately 1.8X on GTX1060 and 1.5X on Tesla V100. Compared to TBE using GA performance models, ROFT achieves an average speed-up ratio of approximately 10X on the Ascend 310 NPU. Specifically, compared to Chameleon and Glimpse on the Nvidia V100, the average speed-up ratios of the tuning time are 1.26X and 0.91X, respectively. As shown in Table 7, except for the case on GTX1060 where the average speed-up ratio is 0.89X that of AutoTVM with XGBoost, the performance is generally consistent, with the average speed-up ratio reaching up to 1.28X.

**Table 6:** Entire network end-to-end tuning time speed-up ratio on GPUs and NPUs.

	VGG-16	MobileNet	ResNet-50	Resnet-18
M1	6.08X	5.77X	–	6.99X
M2	1.87X	1.52X	–	2.10X
M3	7.21X	6.02X	6.08X	–
M4	1.58X	1.83X	1.55X	–
M5	4.83X	3.91X	4.13X	–
M6	12.63X	10.89X	7.44X	–
M7	1.26X	–	–	–
M8	0.91X	–	–	–

M1: compare with AutoTVM with XGBoost on GTX1060. M2: compare with AutoTVM with GA on GTX1060. M3: compare with AutoTVM with XGBoost on Tesla V100. M4: compare with AutoTVM with GA on Tesla V100. M5: compare with AutoTVM with XGBoost on Tesla T4. M6: compare with TBE with GA on Ascend310. M7: compare with Chameleon on Tesla V100. M8: compare with Glimpse on Tesla V100

**Table 7:** Entire network end-to-end performance improvement ratio on GPUs and NPUs.

	VGG-16	MobileNet	ResNet-50	ResNet-18
M1	1.02X	1.00X	–	0.89X
M2	1.31X	1.19X	–	1.02X
M3	1.03X	1.04X	1.02X	–
M4	1.24X	1.25X	1.28X	–
M5	1.05X	1.07X	1.00X	–
M6	1.01X	1.01X	1.01X	–
M7	1.00X	–	–	–
M8	1.00X	–	–	–

M1: compare with AutoTVM with XGBoost on GTX1060. M2: compare with AutoTVM with GA on GTX1060. M3: compare with AutoTVM with XGBoost on Tesla V100. M4: compare with AutoTVM with GA on Tesla V100. M5: compare with AutoTVM with XGBoost on Tesla T4. M6: compare with TBE with GA on Ascend310. M7: compare with Chameleon on Tesla V100. M8: compare with Glimpse on Tesla V100

### Correlation analysis

To further validate the effectiveness of our method, we conducted a correlation analysis on the key metrics. Specifically, we employed Spearman’s rank correlation coefficient^35^ and Kendall’s tau correlation coefficient^35^ to assess the correlation between the average theoretical scores predicted by the ROFT and the empirical scores (based on real executions) observed for each convolutional layer in the VGG-16 network. By using these rank-based correlation methods, we can quantify the monotonic relationship between the theoretical and empirical scores, thereby verifying the consistency and reliability of the algorithm’s optimization results.

Spearman rank correlation coefficient is a nonparametric measure of the monotonic association to investigate the linear relations between two variables. As shown in Table 8, Spearman’s correlation was 0.97 with a p-value of $3.29 \times 10^{-73}$, indicating an extremely strong positive monotonic relationship between the theoretical and empirical scores.

Kendall’s tau correlation coefficient is a non-parametric measure of the association based on the difference between the probabilities of concordance and discordance between two observed variables. As shown in Table 8, Kendall’s Tau was 0.86 with a p-value of $9.29 \times 10^{-44}$, suggesting a high level of rank agreement between the theoretical and empirical scores.

**Table 8:** Correlation analysis of ROFT-predicted theoretical and empirical scores.

	Spearman	Spearman’ P-value	Kendall Tau	Kendall Tau’ P-value
VGG-16	0.97	3.29e−73	0.86	9.29e−44

## Conclusion

In this paper, we propose a Roofline-like cost model and a two-stage search algorithm called ROFT, which significantly accelerate the tuning process of typical operators and DNNs. We implemented the ROFT model using AutoTVM and TBE AutoTune on NVidia’s GPUs and Huawei’s Ascend310 NPUs, respectively. Experimental results on various operators and DNNs demonstrate that ROFT achieves a speed-up of 4X to 10X in the tuning process on GPUs and NPUs, respectively. On the GTX 1060 and Tesla V100, ROFT achieves optimal performance in most cases. The only exceptions are that its tuning time on the V100 is slightly slower than Glimpse, with a speed-up ratio of 0.91X, and its performance on the GTX1060 is slightly lower than AutoTVM with XGBoost, with a speed-up ratio of 0.89X. Experimental results on the GTX 1060 and Tesla V100 for three different networks show that ROFT consistently achieves optimal performance. Using the ROFT algorithm for automatic tuning of common convolutional neural networks in V100, superior performance results can be obtained in a shorter time compared to existing methods. Furthermore, ROFT exhibits certain performance improvements compared to baseline models.From the correlation analysis, both Spearman rank correlation coefficient and Kendall’s Tau correlation coefficient indicate that the theoretical scores predicted by the ROFT model are highly consistent with the empirical measurements, validating the reliability and effectiveness of our optimization method.

## Supplementary Information

Supplementary Information.

## Data Availability

The datasets generated and/or analysed during the current study are available in the uploaded supplementary file ‘ROFT_data.zip’.

## References

[1] Chetlur, S. et al. *cudnn: Efficient Primitives for Deep Learning* (2014).

[2] Chen, T. et al. TVM: An automated End-to-End optimizing compiler for deep learning. In *Proceedings of OSDI 18, 13th USENIX Symposium on Operating Systems Design and Implementation (OSDI 18), Carlsbad, CA*, 578–594 (2018).

[3] Vasilache, N. et al. Tensor comprehensions: Framework-agnostic high-performance machine learning abstractions. arXiv:1802.04730 (2018).

[4] Rotem, N. et al. Glow: Graph lowering compiler techniques for neural networks. arXiv:1805.00907 (2018).

[5] Cyphers, S. et al. Intel ngraph: An intermediate representation, compiler, and executor for deep learning. arXiv:1801.08058 (2018).

[6] Wu, Z. et al. An automated compiler for risc-v based dnn accelerator. In *Proceedings of ISCAS 22, 2022 IEEE International Symposium on Circuits and Systems (ISCAS)*, 3097–3101, 10.1109/ISCAS48785.2022.9937645 (2022).

[7] Xing, J. et al. Bolt: Bridging the gap between auto-tuners and hardware-native performance. *Mach. Learn. Syst.* **4**, 204–216 (2022).

[8] Chen, T. & Guestrin, C. Xgboost: A scalable tree boosting system. In *Proceedings of 22nd acm sigkdd International Conference on Knowledge Discovery and Data Mining*, 785–794 (2016).

[9] Wu, X. et al. Autotuning polybench benchmarks with llvm clang/polly loop optimization pragmas using Bayesian optimization. *Concurr. Comput. Pract. Exp.* **34**, e6683. 10.1002/cpe.6683 (2022).

[10] Robert, S., Zertal, S. & Vaumourin, P. A comparative study of black-box optimization heuristics for online tuning of high performance computing i/o accelerators. *Concurr. Comput. Pract. Exp.* **33**, e6274. 10.1002/cpe.6274 (2021).

[11] Konstantinidis, E. & Cotronis, Y. A quantitative roofline model for gpu kernel performance estimation using micro-benchmarks and hardware metric profiling. *J. Parallel Distrib. Comput.* **107**, 37–56 (2017).

[12] Ragan-Kelley, J. et al. Halide: A language and compiler for optimizing parallelism, locality, and recomputation in image processing pipelines. *Acm Sigplan Not.* **48**, 519–530 (2013).

[13] Kim, T., Kwon, Y., Lee, J., Kim, T. & Ha, S. Cprune: Compiler-informed model pruning for efficient target-aware DNN execution. In *Proceedings of ISCAS 22, Computer Vision–ECCV 2022: 17th European Conference, Tel Aviv, Israel, October 23–27*, 651–667. (Springer, 2022).

[14] Zheng, S. et al. Neoflow: A flexible framework for enabling efficient compilation for high performance DNN training. *IEEE Trans. Parallel Distrib. Syst.* **33**, 3220–3232 (2021).

[15] Ryu, J. & Sung, H. Metatune: Meta-learning based cost model for fast and efficient auto-tuning frameworks (2021).

[16] Baghdadi, R. et al. A deep learning based cost model for automatic code optimization. In *Proceedings of Machine Learning and Systems* (eds Smola, A. et al.), 181–193 (2021).

[17] Gao, X., Wei, C., Zhang, L. & Yang, M. Opevo: An evolutionary method for tensor operator optimization. arXiv:2006.05664 (2020).

[18] Ahn, B. H., Pilligundla, P., Yazdanbakhsh, A. & Esmaeilzadeh, H. Chameleon: Adaptive code optimization for expedited deep neural network compilation. arXiv:2001.08743 (2020). PMC12931927 41743684

[19] Ahn, B. H., Kinzer, S. & Esmaeilzadeh, H. Glimpse: mathematical embedding of hardware specification for neural compilation. In *Proceedings of the 59th ACM/IEEE Design Automation Conference*, 1165–1170 (2022).

[20] Li, M., Zhang, M., Wang, C. & Li, M. Adatune: Adaptive tensor program compilation made efficient. *Adv. Neural Inf. Process. Syst.* **33** (2020).

[21] Yu, C. Improving autotvm efficiency by schedule sharing. (2019).

[22] Unger, C. et al. Unity: Accelerating DNN training through joint optimization of algebraic transformations and parallelization. In *Proceedings of OSDI 22, 16th USENIX Symposium on Operating Systems Design and Implementation (OSDI 22)*, 267–284 (2022).

[23] Borowiec, D., Yeung, G., Friday, A., Harper, R. & Garraghan, P. Doppler: Parallel measurement infrastructure for auto-tuning deep learning tensor programs. *IEEE Trans. Parallel Distrib. Syst.* (2023).

[24] Yang, C.-C., Chen, Y.-R., Liao, H.-H., Chang, Y.-M. & Lee, J.-K. Auto-tuning fixed-point precision with TVM on RISC-V packed SIMD extension. *ACM Trans. Des. Autom. Electron. Syst.* **28**, 1–21 (2023).

[25] Li, M. et al. Exploiting subgraph similarities for efficient auto-tuning of tensor programs. In *Proceedings of the 52nd International Conference on Parallel Processing*, 786–796 (2023).

[26] Wang, Y. et al. Tuna: A static analysis approach to optimizing deep neural networks. arXiv:2104.14641 (2021).

[27] Zheng, L. et al. Ansor: Generating High-Performance tensor programs for deep learning. In *Proceedings of 14th USENIX Symposium on Operating Systems Design and Implementation (OSDI 20)*, 863–879 (2020).

[28] Leinhauser, M. et al. Metrics and design of an instruction roofline model for amd gpus. *ACM Trans. Parallel Comput.* **9**, 1–14 (2022).

[29] Chen, Z. et al. Evaluating performance of AI operators using roofline model. *Appl. Intell.* **52**, 7054–7069 (2022).

[30] Williams, S., Waterman, A. & Patterson, D. Roofline: an insightful visual performance model for multicore architectures. *Commun. ACM* **52**, 65–76 (2009).

[31] Liao, H., Tu, J., Xia, J. & Zhou, X. Davinci: A scalable architecture for neural network computing. In *Proceedings of 2019 IEEE Hot Chips 31 Symposium (HCS)*, 1–44 (IEEE Computer Society, 2019).

[32] Simonyan, K. & Zisserman, A. Very deep convolutional networks for large-scale image recognition. arXiv:1409.1556 (2014).

[33] He, K., Zhang, X., Ren, S. & Sun, J. Deep residual learning for image recognition. In *Proceedings of IEEE Conference on Computer Vision and Pattern Recognition*, 770–778 (2016).

[34] Howard, A. G. et al. Mobilenets: Efficient convolutional neural networks for mobile vision applications. arXiv:1704.04861 (2017).

[35] Essam, F., El, H. & Ali, S. R. H. A comparison of the Pearson, Spearman rank and Kendall tau correlation coefficients using quantitative variables. *Asian J. Probab. Stat* **20**, 36–48 (2022).

