# Supplementary figures and images for: Accelerating the tuning process for optimizing DNN operators by ROFT model
Source: Sci Rep. 2025 Oct 17;15:36327. doi: 10.1038/s41598-025-20139-x (PMC12534627; doi:10.1038/s41598-025-20139-x)

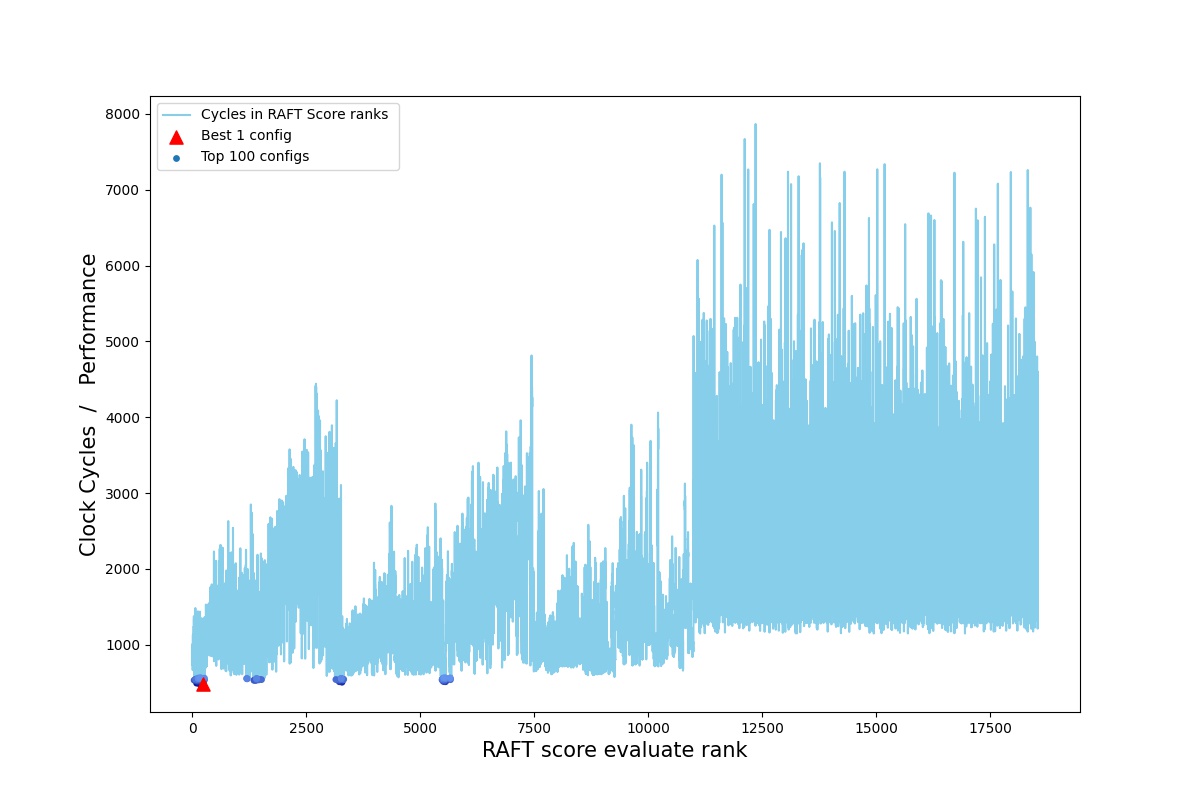

Supplement: Supplementary file 1 — Supplementary Information. [file 41598_2025_20139_MOESM1_ESM.zip › ROFT_data/testcase/offline-log-data/cost_model_conv2d_npu.jpg]
